# Comparison of Different Types of SPME Arrow Sorbents to Analyze Volatile Compounds in *Cirsium setidens* Nakai

**DOI:** 10.3390/foods9060785

**Published:** 2020-06-13

**Authors:** Su-Jeong Kim, Jun-Young Lee, Yun-Sang Choi, Jung-Min Sung, Hae Won Jang

**Affiliations:** Korea Food Research Institute, 245 Nongsaengmyeong-ro, Iseo-myeon, Wanju-Gun, Jeollabuk-do 55365, Korea; sussuu126@gmail.com (S.-J.K.); j.y.lee@kfri.re.kr (J.-Y.L.); kcys0517@kfri.re.kr (Y.-S.C.); jmsung@kfri.re.kr (J.-M.S.)

**Keywords:** *Cirsium setidens* Nakai, volatile compounds, volatile extraction, solid-phase microextraction Arrow, gas chromatography–mass spectrometry

## Abstract

*Cirsium setidens* Nakai is a perennial plant extensively used as food in Korea. Various reports have illustrated the presence of phytochemicals with antioxidant, anti-cancer, anti-tumor, and anti-inflammatory activities; however, little is known about the volatile compounds present in this plant. Here, a novel solid-phase microextraction (SPME) Arrow method was performed to extract and analyze volatile compounds from freeze-dried *Cirsium setidens* Nakai. Four types of SPME Arrows coated with films, such as carbon wide range/polydimethylsiloxane, divinylbenzene/polydimethylsiloxane, polydimethylsiloxane, and polyacrylate were evaluated to identify the most suitable Arrow. The carbon wide range/polydimethylsiloxane Arrow was found to exhibit high affinity for the volatile compounds present in *Cirsium setidens* Nakai. A total of 58 volatile compounds were identified. The major compounds were 2-Pentylfuran, 1-Methylcycloheptanol, 1-Penten-3-ol, 2,2,4,6,6-Pentamethylheptane, 2,3,6,7-Tetramethyloctane, 5-Ethyl-2,2,3-trimethylheptane, 3,5-Octadien-2-one, β-Cyclocitral, and trans-β-Ionone. The present study demonstrates that the SPME Arrow coated with the carbon wide range/polydimethylsiloxane film is suitable for the analytical profiling of volatile compounds present in *Cirsium setidens* Nakai.

## 1. Introduction

*Cirsium setidens* Nakai, a perennial plant belonging to the genus *Cirsium*, is an endemic species of Korea and mainly grows in Kangwon province [1]. It is commonly called “Gondre” in Korea and is extensively used as food and mainly harvested in May, although young leaves and stems of the plant are also consumed in spring. The plant is used in a variety of cooking methods, such as in preparing vegetables, soups, and in fried, stir-fried, and blanched food items [2]. It is also used to prepare tea due to its unique flavor [3].

*C. setidens* Nakai has received increasing attention as a functional food [2]. The *Cirsium* genus, belonging to the Compositae family, contains various bioactive phytochemicals that have various physiological functions, such as antioxidant, anti-cancer, anti-tumor, and anti-inflammatory effects [1,4,5,6]. Pectolinarin, a flavone, is commonly found in plants belonging to the genus *Cirsium*. Pectolinarin and pectolinarigenin, the aglycon of pectolinarin, have hepato-protective and anti-inflammatory activities [7,8]. Silymarin, a polyphenol flavonoid derived from milk thistle, is also present in large amounts in *C. setidens* Nakai. Silymarin is a powerful antioxidant and has various therapeutic properties such as free radical-scavenging, reactive oxygen species-scavenging, and anti-cancer activity [9,10]. *C. setidens* Nakai also contains polyphenols, including pectolinarin and silimarin, in addition to dietary fibers, minerals, and vitamins. Its wide range of physiological activities, due to the presence of a large number of phytochemicals, has been well-studied [3,4].

Recently, the production and consumption of processed food products prepared using *C. setidens* Nakai, such as steamed rice with Gondre, have been steadily increasing due to the unique flavor. However, very little information is available on the volatile compounds from *C. setidens* Nakai, which give the plant its characteristic flavor and taste. Moreover, to the best of our knowledge, only one study has analyzed the volatile compounds in the essential oil extracted from *C. setidens* Nakai via hydrodistillation [11]. To develop food products that can retain the characteristics of *C. setidens* Nakai, further research is required to assess the effect of the processing methods on the taste and flavor of processed food products of *C. setidens* Nakai.

Over the past several decades, numerous studies have been conducted to determine the volatile compounds in food products or their raw materials. The volatile extraction methods such as simultaneous distillation extraction (SDE), solvent-assisted flavor evaporation (SAFE), and steam distillation under reduced pressure (DRP-LLE), which have been used traditionally, have several disadvantages, such as the need for large quantities of extraction solvents and post-extraction solvent evaporation [11,12,13,14,15]. Therefore, headspace extraction methods, such as dynamic headspace, headspace solid-phase microextraction (HS-SPME), and headspace stir bar sorptive extraction (HS-SBSE), are being widely used [15,16,17,18,19,20,21]. The advantages associated with headspace techniques include a simple procedure that avoids the use of organic solvents and high selectivity due to the use of more suitable sorbents, such as polydimethylsiloxane (PDMS), carboxen, divinylbenzene (DVB), and polyacrylate (PA).

**Figure 1:**
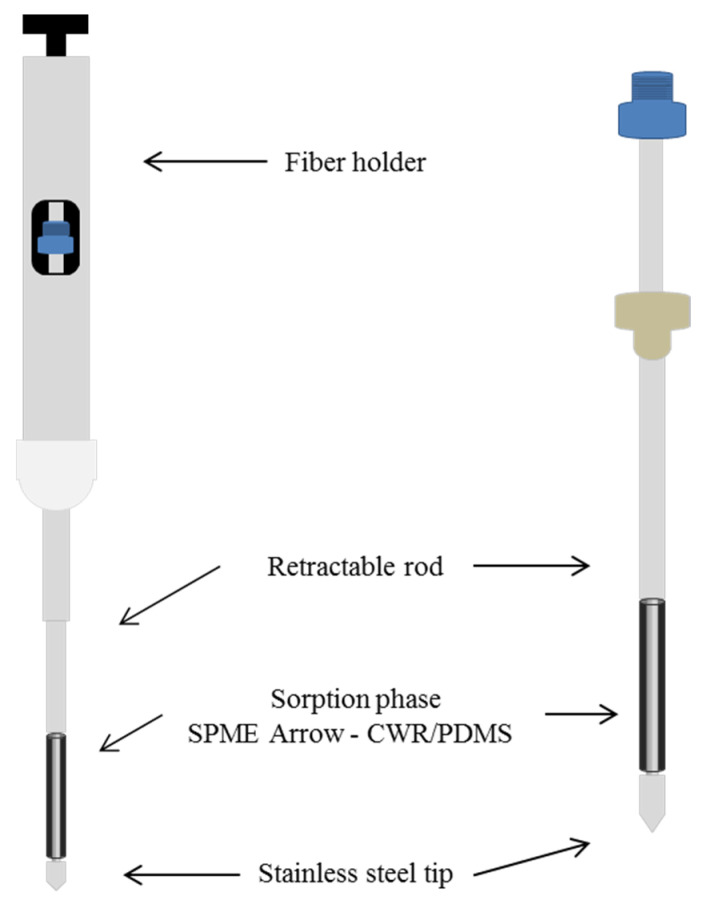
Scheme of solid-phase microextraction (SPME) Arrow device coated with carbon wide range/polydimethylsiloxane (CWR/PDMS).

Recently, the use of an SPME Arrow device, a novel technology that combines the advantages of SPME and HSSE, was introduced for the microextractions of larger volumes of sorbent material compared with that with a traditional SPME fiber [22,23]. This device, consisting of an inner metal rod coated with sorbent materials protected by an outer metal tube, is more robust than the traditional SPME fibers. A scheme of the SPME Arrow device is shown in Figure 1.

SPME Arrows are also covered with a larger volume (6–20 times) of the sorbent phases than SPME fibers, indicating a higher sample capacity [23,24,25]. Therefore, SPME Arrow extracts are more efficient and have better reproducibility than SPME fibers and allow for more volatile compounds to be extracted and analyzed in food [23,24,25]. Since the development of the SPME Arrow, studies on the analysis of harmful components, such as polycyclic aromatic hydrocarbons in water and volatile amines in wastewater, have been carried out [26,27]. However, little research has been reported on the analysis of volatile compounds using SPME Arrows, particularly from food materials. In addition, there is virtually no report on the analysis of volatile compounds from *C. setidens* Nakai using the SPME Arrow system.

In the present study, the volatile compounds of *C. setidens* Nakai were analyzed using four different types of SPME Arrows and compared to determine the most suitable SPME Arrow for the further analysis of *C. setidens* Nakai. We found that the SPME Arrow with a carbon wide range (CWR)/PDMS coating exhibited the highest affinity towards the volatile compounds from the plant. A total of 58 volatile compounds were extracted and identified using gas chromatography–mass spectrometry, amongst which 2-Pentylfuran was found in the highest concentration. Based on our results, we demonstrate that a CWR/PDMS film-coated SPME Arrow is most suitable for the analysis of volatile compounds from *Cirsium setidens* Nakai.

## 2. Materials and Methods

### 2.1. Materials and Reagents

The *C. setidens* Nakai samples used in this study were purchased from a local market in Kangwon province, Korea. Samples were freeze-dried and subsequently ground with dry ice to reduce changes in aromatic ingredients and stored in a freezer below −70 °C until further use. SPME Arrows coated with four different types of films were purchased from CTC Analytics AG (Zwingen, Switzerland) and used to analyze the volatile compounds. The Arrows used were as follows: PDMS (100 μm × 20 mm), PA (100 μm × 20 mm), divinylbenzene/PDMS (DVB/PDMS, 120 μm × 20 mm), and CWR/PDMS (120 μm × 20 mm). In addition, 20-mL headspace screw-top vials and PTFE/silicone septum containing caps for the SPME Arrows were procured from Thermo Fisher Scientific Inc. (West Palm Beach, FL, USA). An alkane standard (C_7_–C_40_) was purchased from Sigma-Aldrich (St. Louis, MO, USA).

### 2.2. Headspace Solid Phase Microextraction Arrow Method

The HS-SPME Arrow was carried out using an RSH Triplus autosampler (Thermo Fisher Scientific Inc., Brookfield, WI, USA). Four different sorbent-coated SPME Arrows were used, and each Arrow was conditioned prior to analysis according to the instructions provided by the manufacturer. For the extraction of volatile components, 1 g of freeze-dried and ground *C. setidens* Nakai samples was placed in 20-mL headspace screw-top vials sealed with PTFE/silicone septum containing caps. The vials were heated and stirred at 50 °C for 10 min to equilibrate the headspace with the volatile compounds in the samples. The Arrows were then exposed to the headspace for 30 min. The volatile compounds in the samples were adsorbed to the sorbent of the respective SPME Arrows, which were then inserted into the injection port in GC and desorbed for 5 min at 250 °C. After desorption, the SPME Arrows were subjected to post-conditioning for 5 min.

### 2.3. Gas Chromatography-Mass Spectrometry (GCMS) Analysis

All samples were separated and detected using a TRACE^™^ 1310 Gas Chromatograph-TSQ 9000 TriPlus quadrupole mass spectrometer (Thermo Fisher Scientific Inc., USA) equipped with a DB-WAX capillary column (60 m × 0.25 mm i.d. × 0.25 μm film thickness) purchased from J & W Scientific Inc., Folsom, CA, USA. The volatile compounds injected into the inlet were delivered to the column at a split ratio of 1:5. Helium was used as a carrier gas with a flow rate of 1 mL/min in a constant flow mode. The GC oven was programed as follows: the initial temperature of 40 °C was maintained for 10 min and then increased to 50 °C at a rate of 2 °C min^−1^. Subsequently, the temperature was further increased from 50 to 100 °C at a rate of 3 °C min^−1^ and then to a final temperature of 210 °C at a rate of 4 °C min^−1^. It was maintained at the final temperature for 5 min. The mass spectrometer was allowed to scan the range from 30 to 500 *m*/*z* in full scan mode. Electron ionization was conducted at 70 eV of ionization energy. The data obtained were processed using Xcalibur. The volatile compounds were identified in two ways that are widely used for profiling volatile compounds. The mass spectra obtained from the GC-MS experiments were compared with the standard spectral data in the Wiley/NIST 2008 library. The Kovats retention index (KI) was calculated using the retention time of the standard n-alkanes and also used for the identification of the peaks. KI was compared with the values in the previously reported literature that can be found in the NIST library, for qualitative analysis. The efficiencies of the four different SPME Arrows were evaluated by comparing the peak areas of each of the volatile components extracted. All experiments were performed in triplicate and expressed as the mean value ± standard deviation.

### 2.4. Statistical Analysis

The results were statistically assessed with an analysis of variance and Duncan’s multiple range test to identify significant differences (*p* < 0.05) using SPSS Statistics ver. 23 (IBM, Armonk, NY, USA).

## 3. Results and Discussion

### 3.1. Selection of SPME Arrow Fibers

**Figure 2:**
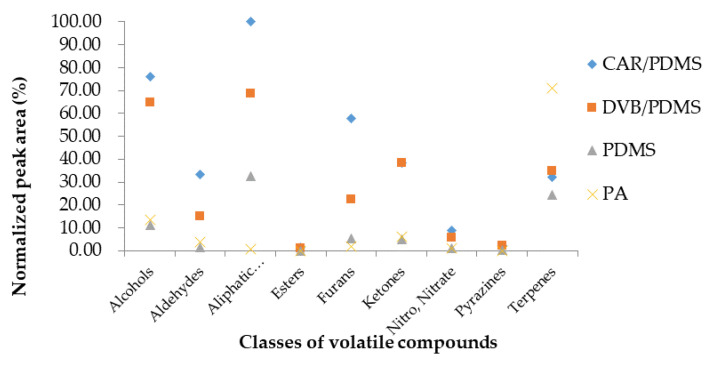
Comparison of the sum of normalized peak areas for each class of volatile compounds extracted from *Cirsium setidens* Nakai using four types of solid-phase microextraction Arrows. CWR/PDMS, carbon wide range/polydimethylsiloxane; DVB, divinylbenzene; PA, polyacrylate.

SPME Arrows can be used to extract the same amounts of volatile compounds twice as fast as SPME fibers [21]. Therefore, this study was conducted using the SPME Arrow method to analyze the volatile compounds present in *C. setidens* Nakai. A total of 58 volatile compounds were extracted and identified using four commercial sorbent-coated (100 μm PDMS, 100 μm PA, 120 μm DVB/PDMS, and 120 μm CWR/PDMS) SPME Arrows. Identified volatile compounds belonged to the following groups: alcohols (6 compounds), aldehydes (12 compounds), aliphatic hydrocarbons (14 compounds), esters (1 compound), furans (4 compounds), ketones (8 compounds), nitro (3 compounds), nitrate (1 compound), pyrazines (2 compound), and terpenes (7 compounds) (Table 1). Figure 2 shows the peak area of each chemical class of the volatile compounds extracted from *C. setidens* Nakai. Our results showed that the extracted volatile compounds were different for the different SPME Arrows varying in the chemical nature of the sorbent. Among the four different sorbent coatings of the SPME Arrows used (CWR/PDMS, DVB/PDMS, PDMS, PA), the highest concentrations of the volatile components were captured using the one coated with CWR/PDMS, as represented by the highest peak area values (10,122.27 × 10^6^). The CWR/PDMS-coated SPME Arrows extracted 52 types of volatile compounds in high amounts as detected from the peaks that appeared. The second highest amount of volatile components was obtained from DVB/PDMS film extraction (peak area value 7322.79 × 10^6^). In contrast, a much lower peak area of the 52 volatile compounds was extracted using the PDMS and PA SPME Arrows. The CWR/PDMS-coated SPME Arrows extracted various types of volatile components that included extractions of components with low KI to high KI, with low-KI components being extracted more efficiently than that with the other SPME Arrows coated with different films. The DVB/PDMS-coated SPME Arrows also extracted volatile compounds with a wide range of polarities. Compared with that of the CWR/PDMS-coated SPME Arrow, the DVB/PDMS-coated SPME Arrow showed a relatively lower extraction of low-KI compounds, such as aldehydes. The SPME Arrow coated with PDMS extracted 37 volatile compounds with peak area values of 2346.02 × 10^6^, whereas the PA-coated SPME Arrow identified only 26 volatile compounds with peak area values of 2831.80 × 10^6^. The smallest number of volatile compounds was extracted using the PA-coated SPME Arrows and the lowest amounts of volatile compounds were extracted using SPME Arrows coated with PDMS. SPME Arrows coated with non-polar PDMS detected most of the aliphatic hydrocarbons with low polarity, such as 2,2,4,6,6-Pentamethylheptane, 2,3,6,7-Tetramethyl octane, and 5-Ethyl-2,2,3-trimethylheptane. Most of the terpenes were also extracted by the PDMS-coated SPME Arrow. However, PDMS was hardly able to extract volatile compounds belonging to other chemical classes and extracted the least number of volatile compounds among the four different sorbents used in this study. Arrows coated with polar films, such as PA, extracted small amounts of volatile compounds. β-Cyclocitral was detected in extremely high abundance from PA fibers; however, aliphatic hydrocarbons with low polarity were detected at very low abundance from PA fibers. Most compounds belonging to the class of aliphatic hydrocarbons, including octane, 2-ethyl-1-hexene, 2,2,6-trimethyloctane, and others, were not detected. Therefore, PA- and PDMS-coated SPME Arrows extracted smaller amounts of volatile compounds compared with those with CWR/PDMS- and DVB/PDMS-coated SPME Arrows.

**Figure 3:**
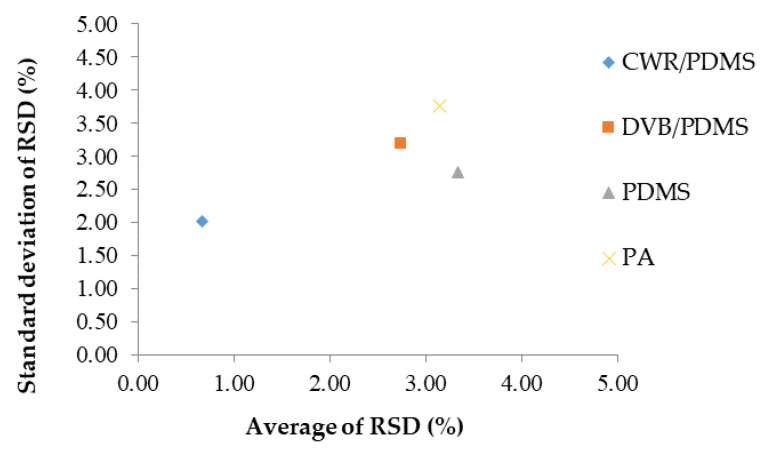
Reproducibility of each solid-phase microextraction Arrow as measured by comparing the relative standard deviation (RSD) (%) and standard deviation of the RSD (%) calculated using identified volatile compounds CWR/PDMS, carbon wide range/polydimethylsiloxane; DVB, divinylbenzene; PA, polyacrylate.

Reproducibility was checked to identify the most suitable sorbent for the SPME Arrows for the analysis of volatile compounds in *C. setidens* Nakai. The relative standard deviation (RSD, %) and standard deviation of the RSD were evaluated for the identified volatile compounds using each of the four SPME Arrows following detection with GC-MS. The results are shown in Figure 3. All four SPME Arrows presented with a low average RSD ranging from 2.01% to 3.76%. These experiments indicated that the CWR/PDMS SPME Arrow is the most suitable to analyze volatile compounds from *C. setidens* Nakai because it covered a wider range of polarity and showed good reproducibility.

### 3.2. Volatile Compounds of Cirsium setidens Nakai

**Figure 4:**
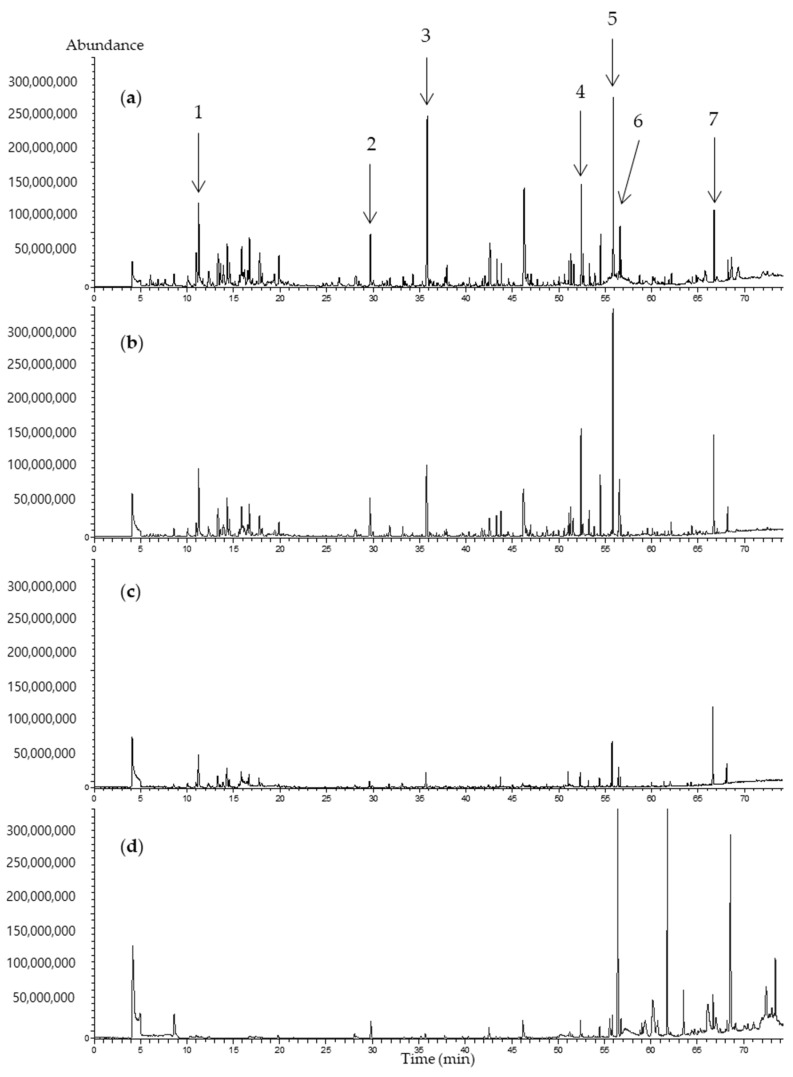
Total ion chromatogram of volatile compounds from *Cirsium setidens* Nakai extracted using solid-phase microextraction Arrows. (**a**) Carbon wide range/polydimethylsiloxane (CWR/PDMS), (**b**) divinylbenzene/PDMS (DVB/PDMS), (**c**) PDMS, and (**d**) polyacrylate (PA). The peak numbers correspond to the compounds as follows: (1) 2,2,4,6,6-Pentamethylheptane, (2) 1-Penten-3-ol, (3) 2-Pentylfuran, (4) 3,5-Octadien-2-one, (5) 1-Methylcycloheptanol, (6) β-Cyclocitral, and (7) trans-β-Ionone.

The chromatograms of the volatile compounds of *C. setidens* Nakai analyzed using the four types of SPME Arrows are shown in Figure 4. Volatile compounds such as 2-Pentylfuran, 2-Ethylfuran, 1-Methylcyloheptanol, 1-Penten-3-ol, 2,2,4,6,6-Pentamethylheptane, 2,3,6,7-Tetramethyloctane, 5-Ethyl-2,2,3-trimethylheptane, 3,5-Octadien-2-one, β-Cyclocitral, and trans-β-Ionone were mainly observed. Other volatile compounds such as caryophyllene, trans-β-Ionone (also named as 4-(2,6,6-Trimethyl-1-cyclohexen-1-yl)-3-buten-2-one), and β-Ionone epoxide were previously reported in the essential oils extracted from *C. setidens* Nakai [11]. Volatile compounds belonging to the alcohol group were present in large amounts based on the peak areas (from 327.16 × 10^6^ to 2200.41 × 10^6^ area units) in the extractions performed from all four types of SPME Arrows. In addition, aliphatic hydrocarbons, especially 2,2,4,6,6-pentamethylheptane, were the most abundant volatile compounds extracted by all four SPME Arrows, except the PA-coated Arrow. Further, 1-Methylcycloheptanol, 1-Penten-3-ol, and *trans*-β-Ionone were also present in high amounts based on the peak area in the extractions from all four Arrows. Among those compounds, 1-Penten-3-ol is known to smell like green vegetables [28] and *trans*-β-ionone represents a violet-like, sweet, and floral scent [29,30]. In particular, volatiles extracted with CWR/PDMS-coated SPME Arrows showed a particularly high amount of 2-pentylfuran, which is formed from linoleic acid and is well-known for its involvement in lipid oxidation. It is reported to smell like earthy beany vegetables and is expected to undergo changes in content upon drying and storage depending upon storage conditions, which can eventually change the flavor [31,32]. Moreover, 3,5-Octadien-2-one, which imparts a mushroom-like flavor, was also present in high amounts [33]. β-Cyclocitral was present in high amounts based on the peak area in the extractions performed by all SPME Arrows except that with a PDMS coating. β-Cyclocitral is one of the monoterpenoids derived from carotenoids. It is an important compound, commonly found in many plants, vegetables, and fruits, has a sweet smell, and imparts a distinct fruity flavor [33]. The presence of the volatile compounds that belong to the group of terpenes are consistent with previous reports by Choi that demonstrated the presence of these compounds in essential oils obtained from *C. setidens* [11].

## 4. Conclusions

SPME has been widely used to analyze the volatile compounds of food materials. More recently, SPME Arrows were developed as an analytical tool that improved upon the SPME fiber with respect to robustness, sensitivity, and sorbent phase surfaces and volumes. However, few analytical studies using SPME Arrows have been reported. Here, we analyzed the volatile components of *C. setidens* Nakai, a plant native to Korea, using the SPME Arrow method. Four types of SPME Arrows were analyzed and their efficiency to extract volatile compounds was compared by analyzing the profiles of volatile compounds extracted from the plant samples. A total of 58 components were extracted and identified. The extracted volatile compounds belonged to several chemical groups that included alcohols (6 compounds), aldehydes (12 compounds), aliphatic hydrocarbons (14 compounds), esters (1 compound), furans (4 compounds), ketones (8 compounds), nitro and nitrates (4 compounds), pyrazines (2 compounds), and terpenes (7 compounds). The major compounds obtained by using four types of SPME Arrows were 2-Pentylfuran, 1-Methylcyloheptanol, 1-Penten-3-ol, 2,2,4,6,6-Pentamethylheptane, 2,3,6,7-Tetramethyloctane, 5-Ethyl-2,2,3-trimethylheptane, 3,5-Octadien-2-one, β-Cyclocitral, and trans-β-Ionone. The aromatic compounds with varied odors were further investigated using the four SPME Arrows. It was revealed that the volatile compounds were extracted differently according to the chemical properties of the sorbent materials used in the Arrows, which provided them with specific affinities for the volatile compounds. The results showed that the use of SPME Arrows with the CWR PDMS and DVB/PDMS sorbents resulted in high volatile extraction efficiency. In particular, the CWR/PDMS-coated SPME Arrow extracted various components with high efficiency. Therefore, based on our results, it could be considered suitable for profiling the volatile compounds from *C. setidens* Nakai. To develop food products and functional foods using *C. setidens* Nakai, further research is required to monitor the changes that occur in the chemical compositions of the volatile compounds depending upon the processing methods used for the preparation of food products. Our study outcomes show that the CWR/PDMS-coated SPME Arrow could be used to carry out future studies to gain insights into the effects of processing methods on the chemical compositions of volatile compounds present in food products that impart them with distinct flavors and tastes.

## Figures and Tables

**Table 1:** Volatile compounds from freeze-dried *Cirsium setidens* Nakai analyzed using different types of solid-phase microextraction Arrows.

Compounds	ID ^a^	KI ^b^	KI ^c^	Peak Area (× 10^6^, mean ± SD) ^d^			
				CWR/PDMS	DVB/PDMS	PDMS	PA
Alcohols							
1-Penten-3-ol	MS, KI	1172	1165	479.54 ± 1.84 ^a^	333.05 ± 2.49 ^b^	53.74 ± 0.10 ^d^	153.77 ± 3.08 ^c^
1-Pentanol	MS, KI	1260	1261	83.73 ± 2.17 ^a^	52.70 ± 0.09 ^b^	ND	16.84 ± 0.06 ^c^
(Z)-2-Penten-1-ol	MS, KI	1327	1325	369.49 ± 6.31 ^a^	154.93 ± 1.34 ^b^	15.60 ± 1.06 ^d^	82.52 ± 1.92 ^c^
1-Hexanol	MS, KI	1361	1359	76.50 ± 2.12 ^a^	35.61 ± 1.11 ^b^	ND	8.60 ± 0.11 ^c^
1-Octen-3-ol	MS, KI	1456	1456	30.88 ± 1.08 ^a^	26.91 ± 0.00 ^b^	4.66 ± 0.42 ^c^	ND
1-Methylcycloheptanol	MS	1598		1160.26 ± 75.40 ^b^	1270.00 ± 31.02 ^a^	253.16 ± 4.04 ^c^	126.27 ± 7.96 ^d^
Aldehydes							
2-Methylbutanal	MS, KI	912	896	ND	7.18 ± 0.35	ND	ND
3-Methylbutanal	MS, KI	916	900	ND	5.96 ± 0.03	ND	ND
Hexanal	MS, KI	1078	1081	273.84 ± 6.57 ^a^	109.82 ± 1.32 ^b^	ND	23.44 ± 1.21 ^c^
(E)-2-Pentenal	MS, KI	1130	1130	33.92 ± 0.10 ^a^	ND	ND	5.92 ± 0.27 ^b^
2-Methyl-2-pentenal	MS, KI	1162	1149	55.26 ± 0.85 ^a^	17.29 ± 0.96 ^b^	ND	ND
Heptanal	MS, KI	1186	1186	51.11 ± 0.18 ^a^	20.55 ± 0.65 ^b^	ND	ND
2-Hexenal	MS, KI	1214	1207	101.18 ± 0.26 ^a^	32.46 ± 0.12 ^b^	ND	7.28 ± 0.37 ^c^
(Z)-4-Heptenal	MS, KI	1244	1247	29.54 ± 0.70	ND	ND	ND
(E)-2-Heptenal	MS, KI	1324	1318	24.33 ± 0.31 ^a^	ND	ND	ND
(E)-2-Octenal	MS, KI	1432	1434	22.83 ± 0.49 ^a^	ND	6.80 ± 0.04 ^b^	ND
(E,E)-2,4-Heptadienal	MS, KI	1495	1497	174.35 ± 6.61 ^a^	158.03 ± 3.00 ^b^	28.78 ± 0.55 ^d^	48.63 ± 1.52 ^c^
Benzaldehyde	MS, KI	1528	1528	194.50 ± 10.69 ^a^	81.81 ± 0.24 ^b^	11.01 ± 0.31 ^c^	18.96 ± 1.69 ^c^
Aliphatic hydrocarbons							
Octane	MS, KI	800	800	68.36 ± 3.37 ^a^	12.67 ± 0.50 ^b^	4.80 ± 0.11 ^c^	ND
2-Ethyl-1-hexene	MS, KI	837	847	60.74 ± 3.41 ^a^	12.22 ± 0.32 ^b^	5.45 ± 0.38 ^c^	ND
2,2,6-Trimethyloctane	MS	935		154.78 ± 4.50 ^a^	80.45 ± 0.17 ^b^	37.38 ± 0.92 ^c^	ND
2,2,4,6,6-Pentamethylheptane	MS, KI	959	957	857.19 ± 15.49 ^a^	647.06 ± 5.08 ^b^	321.92 ± 5.27 ^c^	8.07 ± 0.06 ^d^
3-Methylnonane	MS, KI	966	965	58.53 ± 2.18 ^a^	16.88 ± 0.04 ^b^	5.46 ± 0.10 ^c^	ND
Decane	MS, KI	999	1000	200.37 ± 1.59 ^a^	60.33 ± 0.43 ^b^	21.50 ± 0.07 ^c^	ND
2,3,6,7-Tetramethyloctane	MS	1009		425.57 ± 6.09 ^a^	388.08 ± 0.87 ^b^	204.57 ± 0.84 ^c^	ND
5-Ethyl-2,2,3-trimethylheptane	MS	1031		387.76 ± 7.27 ^a^	289.75 ± 0.99 ^b^	158.50 ± 4.31 ^c^	ND
2,2,7,7-Tetramethyloctane	MS	1042		414.62 ± 5.55 ^a^	274.51 ± 5.57 ^b^	117.66 ± 0.12 ^c^	ND
3-Methylundecane	MS	1175		62.80 ± 3.71 ^a^	45.28 ± 1.63 ^b^	16.16 ± 0.46 ^c^	ND
Dodecane	MS	1199		75.18 ± 1.07 ^a^	65.48 ± 1.76 ^b^	27.26 ± 0.08 ^c^	ND
3-Methylene-undecane	MS	1240		40.55 ± 0.15 ^a^	31.45 ± 0.29 ^b^	ND	ND
3-Methyltridecane	MS	1370		24.35 ± 0.25 ^a^	20.48 ± 0.87 ^b^	11.94 ± 0.52 ^c^	ND
(Z)-1,4-Hexadiene	MS	1560		59.38 ± 0.89 ^a^	45.44 ± 0.21 ^b^	3.99 ± 0.22 ^d^	11.82 ± 0.15 ^c^
Esters							
Ethyl hexanoate	MS, KI	1239	1241	44.46 ± 1.73 ^a^	31.23 ± 0.12 ^b^	ND	1.96 ± 0.09 ^c^
Furans							
2-Methylfuran	MS, KI	868	876	51.30 ± 0.26 ^a^	13.97 ± 0.11 ^b^	5.04 ± 0.51 ^c^	ND
2-Ethylfuran	MS, KI	954	960	316.11 ± 3.12 ^a^	124.42 ± 0.69 ^b^	48.70 ± 0.32 ^c^	23.52 ± 2.05 ^d^
2-Pentylfuran	MS, KI	1234	1235	1266.54 ± 32.31 ^a^	487.03 ± 0.65 ^b^	98.17 ± 0.44 ^c^	29.81 ± 1.25 ^d^
trans-2-(2-Pentenyl)furan	MS	1300		33.72 ± 1.16 ^a^	20.27 ± 0.03 ^b^	ND	ND
Ketones							
2-Heptanone	MS, KI	1184	1185	25.74 ± 0.13 ^a^	10.68 ± 0.10 ^b^	ND	ND
Acetoin	MS, KI	1283	1287	22.56 ± 0.80 ^a^	22.71 ± 2.19 ^a^	ND	12.63 ± 0.49 ^b^
2,2,6-Trimethylcyclohexanone	MS, KI	1312	1307	50.79 ± 0.53 ^b^	53.09 ± 0.31 ^a^	11.66 ± 0.17 ^c^	3.39 ± 0.09 ^d^
6-Methyl-6-hepten-2-one	MS, KI	1318	1320	72.19 ± 1.10 ^a^	37.42 ± 1.18 ^b^	ND	8.00 ± 0.15 ^c^
6-Methyl-5-heptene-2-one	MS, KI	1340	1341	161.23 ± 1.83 ^a^	118.61 ± 0.30 ^b^	14.05 ± 0.41 ^c^	8.83 ± 0.10 ^d^
3,5-Octadien-2-one	MS, KI	1524	1516	534.74 ± 0.51 ^a^	534.56 ± 15.23 ^a^	76.33 ± 0.10 ^c^	87.85 ± 3.52 ^b^
Ketoisophorone	MS, KI	1704	1708	ND	30.05 ± 1.63	ND	ND
Nitro, Nitrate							
2-Nitropropane	MS	1202		49.58 ± 1.11 ^a^	25.75 ± 0.07 ^b^	ND	11.16 ± 0.18 ^c^
3-Methyl-1-butanol nitrate	MS	1228		41.11 ± 0.78 ^a^	19.96 ± 0.12 ^b^	ND	ND
1-Nitrobutane	MS, KI	1291	1310	58.69 ± 0.02 ^a^	32.46 ± 1.16 ^b^	9.84 ± 1.11 ^c^	8.71 ± 0.30 ^d^
1-Nitrohexane	MS, KI	1501	1502	108.95 ± 1.00 ^a^	90.19 ± 1.70 ^b^	15.65 ± 0.31 ^c^	15.74 ± 0.04 ^c^
Pyrazines							
Trimethylpyrazine	MS, KI	1406	1413	24.13 ± 2.14 ^a^	22.86 ± 0.33 ^b^	ND	ND
Tetramethylpyrazine	MS, KI	1481	1467	39.26 ± 2.87 ^a^	35.71 ± 2.23 ^b^	10.16 ± 0.17 ^c^	ND
Terpenes							
Caryophyllene	MS, KI	1608	1598	81.57 ± 4.56 ^a^	30.15 ± 2.80 ^b^	17.69 ± 1.67 ^c^	ND
β-Cyclocitral	MS, KI	1622	1600	304.70 ± 1.82 ^c^	311.87 ± 6.94 ^b^	106.14 ± 1.46 ^c^	1846.12 ± 56.83 ^a^
β-Selinene	MS, KI	1738	1725	ND	ND	23.12 ± 0.45	ND
Geranylacetone	MS, KI	1864	1865	ND	15.75 ± 0.55 ^a^	13.86 ± 0.62 ^b^	ND
α-Ionone	MS, KI	1868	1857	ND	ND	0.05 ± 0.00	ND
trans-β-Ionone	MS, KI	1961	1954	389.65 ± 1.50 ^c^	506.51 ± 24.73 ^a^	428.17 ± 24.52 ^b^	204.47 ± 24.73 ^d^
β-Ionone epoxide	MS, KI	2015	2002	150.59 ± 7.95 ^a^	144.34 ± 6.67 ^b^	108.47 ± 2.14 ^c^	ND

All values are represented by the mean ± standard deviation (*n* = 3). KI, Kovats retention index; CWR/PDMS, carbon wide range/polydimethylsiloxane; DVB, divinylbenzene; PA, polyacrylate. ^a^ Identification method, MS: confirmed by matching the mass spectrum gained experimentally and mass spectrum in the Wiley and NIST libraries, KI: identified by comparing KI ^b^ and KI ^c^. ^b^ KI calculated using *n*-alkanes for DB-WAX column. ^c^ KI reported from NIST available at http://webbook.nist.gov/chemistry/cas-ser.html for DB-WAX columns or equivalents. ^d^ Peak area values expressed by ×10^6^. ND, not detected.
